# Dynamics of symbiont-mediated antibiotic production reveal efficient long-term protection for beewolf offspring

**DOI:** 10.1186/1742-9994-10-3

**Published:** 2013-01-31

**Authors:** Sabrina Koehler, Jan Doubský, Martin Kaltenpoth

**Affiliations:** 1 Max Planck Institute for Chemical Ecology, Insect Symbiosis Research Group, Hans-Knoell-Str. 8, 07745, Jena, Germany; 2 Max Planck Institute for Chemical Ecology, Mass Spectrometry/Proteomics Research Group, Jena, Germany; 3 Present address: API Synthesis Department, Zentiva, Prague, Czech Republic

**Keywords:** Defensive symbiosis, Protective mutualism, Crabronidae, Antibiotic, *Streptomyces*, Hymenoptera, Streptochlorin synthesis

## Abstract

**Background:**

Insects have evolved a wide range of mechanisms to defend themselves and their offspring against antagonists. One of these strategies involves the utilization of antimicrobial compounds provided by symbiotic bacteria to protect the host or its nutritional resources from pathogens and parasites. In the symbiosis of the solitary digger wasp, *Philanthus triangulum* (Hymenoptera, Crabronidae), the bacterial symbiont ‘*Candidatus* Streptomyces philanthi’ defends the developing larvae against pathogens by producing a mixture of at least nine antimicrobial substances on the cocoon surface*.* This antibiotic cocktail inhibits the growth of a broad range of detrimental fungi and bacteria, thereby significantly enhancing the offspring’s survival probability.

**Results:**

Here we show that the production of antimicrobial compounds by the beewolf symbionts is confined to the first two weeks after cocoon spinning, leading to a high concentration of piericidins and streptochlorin on the cocoon surface. Expression profiling of housekeeping, sporulation, and antibiotic biosynthesis genes indicates that antibiotic production coincides with morphological differentiation that enables the symbionts to survive the nutrient-limited conditions on the beewolf cocoon. The antibiotic substances remain stable on the cocoon surface for the entire duration of the beewolf’s hibernation period, demonstrating that the compounds are resistant against environmental influences.

**Conclusions:**

The antibiotic production by the beewolf symbionts serves as a reliable protection for the wasp offspring against pathogenic microorganisms during the long and unpredictable developmental phase in the subterranean brood cells. Thus, the beewolf-*Streptomyces* symbiosis provides one of the rare examples of antibiotics serving as an efficient defense in the natural environment and may aid in devising new strategies for the utilization of antibiotic combination therapies in human medicine against increasingly resistant bacterial and fungal pathogens.

## Background

Predators, parasites and pathogens can significantly reduce the reproductive success of insects. Developmental stages are especially prone to pathogen infestation given their often limited mobility as well as trade-offs that lead to limited resources being allocated into growth instead of defense [1-4]. Additionally, many insect species rear their offspring in subterranean nesting sites where the progeny is continuously threatened by soil pathogens [5-7]. To counteract these threats, insects have evolved an intricate immune system as well as chemical and behavioral defenses, including brood care, nesting in habitats which are difficult to invade for predators or brood parasites, and the application of antimicrobial substances as defense against pathogen infestation [7-10].

Sociality has been documented to provide insects with collective defensive behaviors aimed at eliminating parasites from group members and thereby limiting the spread of infections within the colony [11-14]. Specifically, allogrooming can serve as an efficient strategy to mechanically remove fungal spores, nematodes, mites and other parasites [11,15,16]. By contrast, solitary species are usually lacking these behavioral defenses [14,17-19], making other means of protection of their offspring against fungal and bacterial pathogens particularly important. Although nest hygienic behaviors have also been observed in solitary insects [19], there is often no or only limited contact between adult and developing individuals, so chemical mechanisms are likely to play a more important role for antimicrobial defense [7,20-22]. Concordantly, the production and use of various defensive compounds for the protection of the developing offspring has been described for a number of solitary insect species [8,23-28].

In addition to the insect’s own defenses, recent studies have demonstrated that several taxa team up with bacterial symbionts to protect the host, the offspring or its nutritional resources against pathogens, predators, parasites, or parasitoids [20,21,29-33]. This protection can be mediated by (i) competitive exclusion of pathogenic microorganisms [34-36], (ii) interaction with the host’s immune system to enhance resistance against pathogenic infestation [17,37,38], or (iii) the production of chemicals that harm and/or deter antagonists [29,33,39-41].

Interestingly, many of the mutualistic microorganisms involved in defensive partnerships with insects belong to the bacterial phylum Actinobacteria [42,43]. Members of this group appear to be predisposed towards engaging in defensive symbioses by their widespread distribution in the soil, the ability to subsist in nutritionally deficient environments, and, notably, their capacity to produce a wide variety of secondary metabolites with antimicrobial properties [40,42,44,45]. These compounds may provide an efficient way to defend the producer against pathogenic or competing microorganisms, making Actinobacteria particularly suitable partners for defensive mutualistic interactions with insect hosts. Especially species of the genus *Streptomyces* are efficient producers of antimicrobial substances, with over two-thirds of the clinically relevant natural product antibiotics originating from this genus [44,46]. Despite the common perception of antibiotics as agents of chemical warfare among competing microorganisms, evidence for the natural roles of these compounds and their defensive activity under *in vivo* conditions is still scarce [47,48]. In fact, recent studies suggest that their primary effect in maintaining microbial communities may be the modulation of gene transcription at low concentrations rather than the elimination of competitors [49].

Fungus-farming ants represent a prime example of protective symbioses. The ants cultivate symbiotic fungi as a food source for their colony. To protect their source of nourishment from fungal infestation by the pathogenic fungus *Escovopsis*, the ants engage in a protective symbiosis with actinomycete bacteria which have been reported to produce antibiotic substances that inhibit the growth of *Escovopsis*[30,41,50-52], thereby protecting the fungal gardens without affecting the cultivar itself [53]. Similarly, the fungus growing bark beetle *Dendroctonus frontalis* is associated with bacteria of the genus *Streptomyces*[33]*.* The bacteria, present in the fungal galleries as well as in the mycangia of the beetle, produce the antifungal substance mycangimycin, which can protect the beetle’s nutritional resources against the antagonistic fungus *Ophiostoma minus*[33,39].

Solitary digger wasps in the tribe Philanthini (‘beewolves’, Hymenoptera: Crabronidae) engage in a defensive symbiosis with bacteria of the genus *Streptomyces* for the protection of the developing offspring [20,40,54,55]. Female beewolves cultivate the symbiotic microorganisms in specialized antennal gland reservoirs [56] and secrete them into the brood cells prior to laying an egg on one of the provisioned bees [20]. After oviposition, the female seals the brood cell with sand and subsequently does not provide any further brood care. As beewolf development can take up to nine months, including a long period of larval diapause during hibernation in the humid underground brood cells, the beewolf’s offspring is continuously threatened by pathogenic bacteria and fungi that may invade the brood cells from the surrounding soil as well as from the remains of the provisioned honeybee prey [57]. As an efficient broad-spectrum defense, the larvae incorporate the symbiotic streptomycetes into the silken walls of their cocoons, where the symbionts produce at least nine different antibiotic substances that serve as an “antimicrobial combination prophylaxis” against pathogenic bacteria and fungi during the long and vulnerable period of hibernation [40].

The mutualistic association of beewolves and *Streptomyces* provides a unique opportunity to study the natural role of antimicrobial compounds in a symbiotic context [20,40,54,58]. Here we investigated the dynamics of population size and antibiotic production of the symbiotic *Streptomyces* on the beewolf cocoon in order to elucidate the mechanisms that allow for the long-term defense against pathogens. The results yield insights into an efficient strategy for offspring protection in a solitary insect and provide a unique case study on the long-term efficacy of bacterial secondary metabolites directly in the natural environment.

## Results

### *Streptomyces* population dynamics on beewolf cocoons

**Figure 1:**
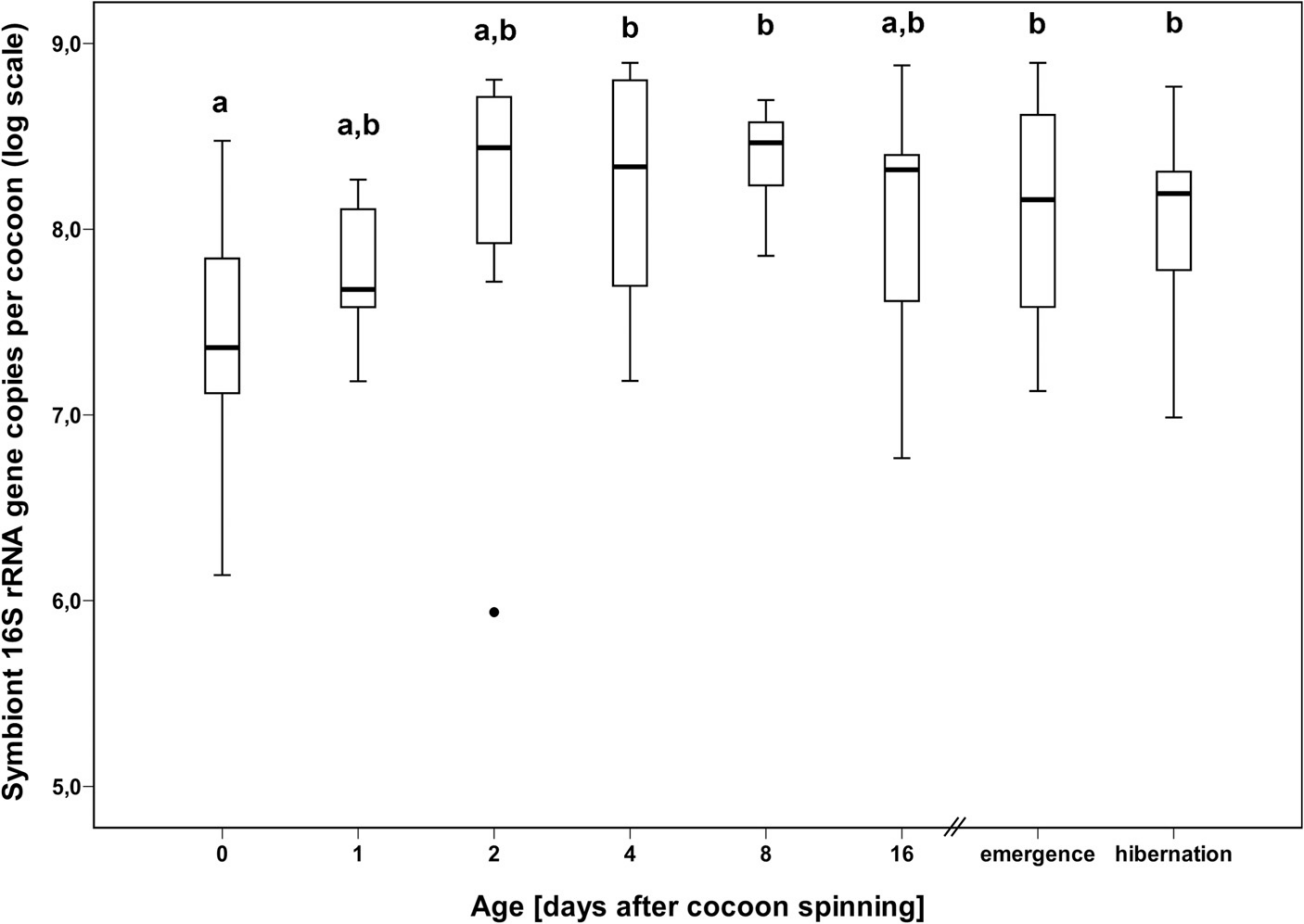
**Estimated 16S rRNA gene copy number of *Streptomyces *bacterial cells on male beewolf cocoons of different ages after cocoon spinning (on log scale; the different groups refer to the days after cocoon spinning). **Bold lines represent medians obtained from qPCR of 16S rRNA, boxes comprise the interquartile range, and bars indicate minimum and maximum values, outliers are given as dots. Different letters above the boxes represent significant differences (ANOVA with Tukey post-hoc tests: df=7, p=0.02).

The number of *Streptomyces* bacterial cells was quantified for different time points after cocoon spinning (day 0 [n=11], 1 [n=7], 2 [n=7], 4 [n=25], 8 [n=27], 16 [n=9], emergence without hibernation [n=32], emergence after hibernation [n=39]) using quantitative real-time PCR (qPCR) analysis of the 16S rRNA gene. ‘Emergence without hibernation’ refers to beewolves that emerged from the cocoon about four weeks after cocoon spinning without entering diapause, whereas ‘emergence after hibernation’ designates cocoons of individuals that entered diapause and were kept for eight months at 6°C before completing development and emerging from the cocoon (for details see Methods section). Thus, the symbiont numbers on cocoons in the two ‘emergence’ groups represent the population sizes present after the beewolves completed development. The estimated 16S copy numbers (representing the total values per beewolf cocoon) indicate that the symbiont population size increases within the first two days after cocoon spinning followed by cessation of bacterial growth (Figure 1; ANOVA: df=7, p=0.02).

### Dynamics of the antibiotic cocktail and piericidin gene expression

**Figure 2:**
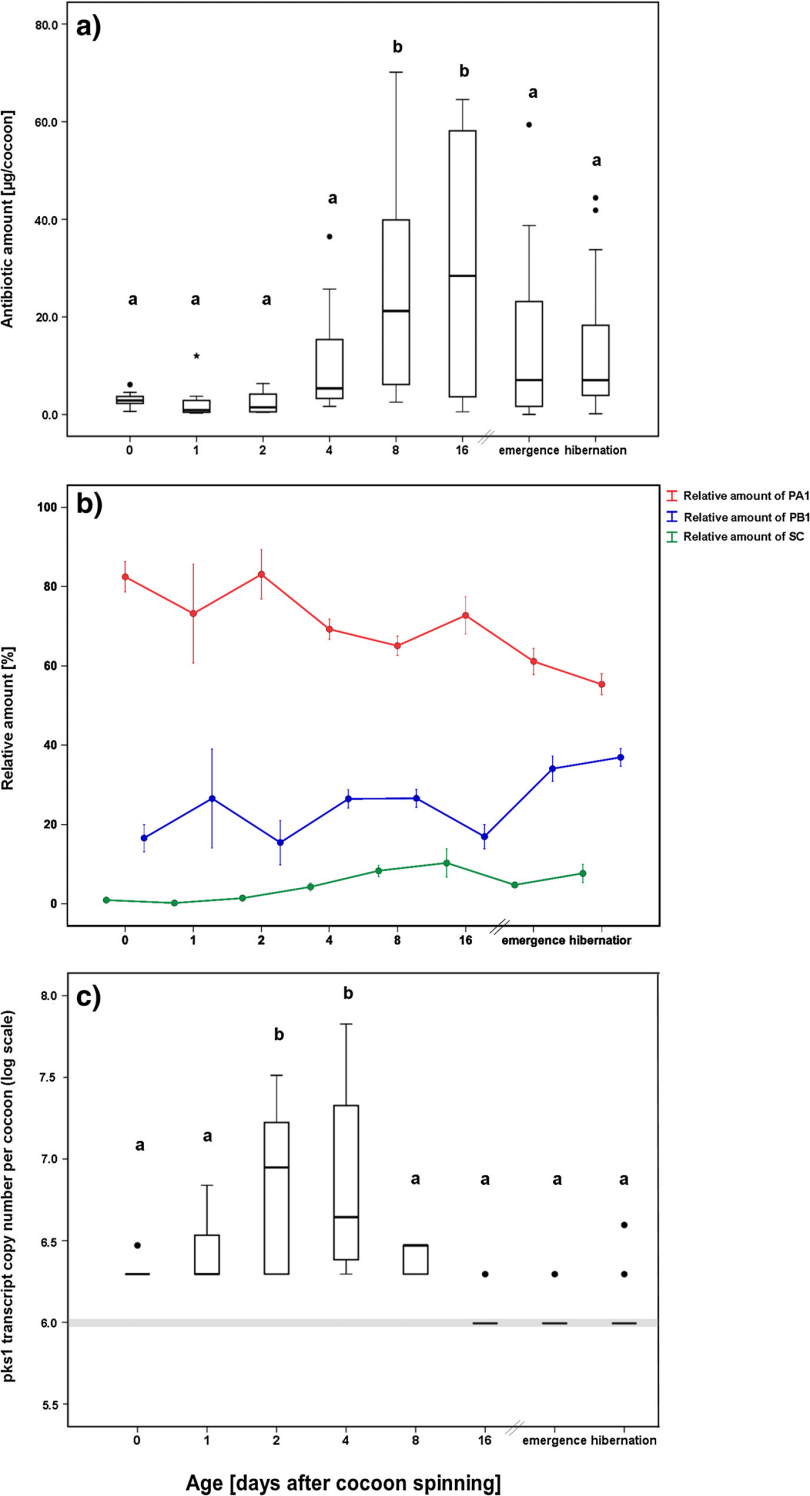
**Dynamics of antibiotic production on beewolf cocoons. a)** Total amount [μg/cocoon] of antibiotic substances on beewolf cocoons at different time points after cocoon spinning quantified using GC-MS. Different letters above the boxes represent significant differences (ANOVA with Tukey post-hoc tests: df=7, p<0.001), **b)** Average relative amounts of the three most abundant antibiotic substances on beewolf cocoons (in %) at different time points after cocoon spinning quantified by GC-MS. A discriminant analysis based on the composition of the antibiotic cocktail correctly classified 20–72.2% of the originally grouped cases (40.1% on average, Wilks’ Lambda: 0.432, Chi^2^: 126.4, df=21, p<0.001, see Additional file 1: Figure S1), **c)** Absolute number of putative piericidin transcripts (*pks1*) on beewolf cocoons at different time points after cocoon spinning, obtained from RT-qPCR assays (on log scale). Different letters above the boxes represent significant differences (ANOVA with Tukey post-hoc tests: df=7, p<0.001). The different groups refer to days after cocoon spinning; emergence: eclosion of adult beewolf from the cocoon (about four weeks after cocoon spinning), hibernation: eclosion after diapause (about 8 months after cocoon spinning). Bold lines represent medians, boxes comprise the interquartile range, bars indicate minimum and maximum value, outliers are given as dots; the grey bar indicates the level of unspecific amplification as obtained from the qPCR negative control.

Gas chromatography – mass spectrometry (GC-MS) analyses of methanol extracted beewolf cocoons at different time points after cocoon spinning showed significant changes in the amount of the major antibiotics on the cocoon over time (Figure 2a, ANOVA: df=7, p<0.001). Directly after cocoon spinning (Day 0–2), there was already a low amount of antibiotics (0.6–4.5 μg/cocoon) present on the cocoon surface. Within the following 1–2 weeks, the amount of antibiotics slowly increased, with an average amount of 26.4 μg/cocoon at day 8 to 16, and subsequently decreased slowly over time. Nevertheless, there was still a considerable amount of antibiotics present on the surface of beewolf cocoons after hibernation for approximately nine months, with an average amount of 12.6 μg/cocoon.

The discriminant analysis based on the relative amounts of streptochlorin (SC), piericidin A1 (PA1), and piericidin B1 (PB1) showed a significant change in the composition of the antibiotic cocktail over time, suggesting temporal differences in the production and/or deterioration of the single compounds (Wilks’ Lambda=0.432, Chi^2^=126.4, df=21, p<0.001; Figure 2b, Additional file 1: Figure S1). In particular, we detected a decline in the relative amount of PA1 as compared to SC and PB1 towards the later time points, suggesting that production of PA1 starts earlier than that of SC and PB1, and that PA1 may be less stable than the other two compounds.

The expression of the putative piericidin gene (*pks1*) revealed a similar pattern as the chemical analyses, albeit shifted to earlier time points. Expression levels peaked at day 2 and 4 and subsequently decreased to background levels (Figure 2c, ANOVA: df=7, p<0.001).

### Expression of housekeeping and sporulation genes

**Figure 3:**
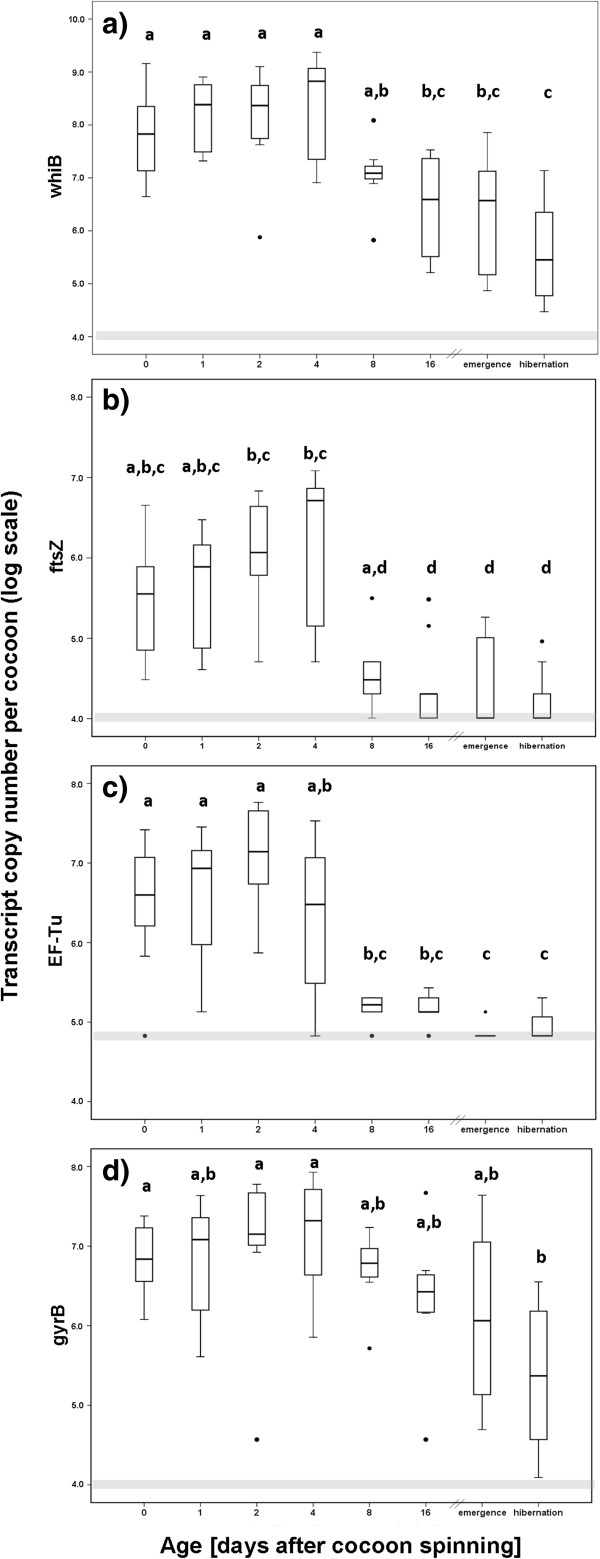
**Expression levels of different housekeeping genes and the *whiB *sporulation regulatory gene on beewolf cocoons at different time points after cocoon spinning, obtained from RT-qPCR assays (on log scale). a)***whiB*; **b)***ftsZ*; **c)***EF-Tu*; **d)***gyrB*. The different groups refer to days after cocoon spinning; emergence: eclosion of adult beewolf from the cocoon (about four weeks after cocoon spinning), hibernation: eclosion after diapause (about 8 months after cocoon spinning). Bold lines represent medians, boxes comprise the interquartile range, bars indicate minimum and maximum values, outliers are given as dots. Different letters above the boxes represent significant differences (ANOVA with Tukey post-hoc tests: df=7, p<0.05 for all analyses), The grey bar indicates the level of unspecific amplification as obtained from the qPCR negative control.

The expression of the housekeeping genes *ftsZ* and *EF-Tu* as well as the sporulation regulatory gene *whiB*, obtained from qPCR analyses, remained constant for the first four days after cocoon spinning (Figure 3a-c). However, at day 8 the expression level of all three genes decreased by several orders of magnitude, indicating a reduced metabolism of the *Streptomyces* symbionts approximately one week after cocoon spinning, probably due to morphological differentiation. By contrast, the transcript level of *gyrB* decreased more slowly over time, suggesting a role of the gyrase topoisomerases during the onset of the dormant phase (Figure 3d).

## Discussion

An improved understanding of the production and ecology of antimicrobial substances in natural environments is important to complement our present knowledge on the evolution of antibiotic resistance and to acquire new perspectives for the utilization of antibiotics in human medicine. The beewolf-*Streptomyes* symbiosis represents a unique model system to study these factors *in vivo*. Our results indicate that an “antibiotic cocktail” is produced by the defensive *Streptomyces* symbionts on beewolf cocoons within the first two weeks of larval development and subsequently serves as a reliable long-term antimicrobial protection for the wasp offspring. The stability and long-term efficacy of the symbiosis is mediated by morphological differentiation of the bacteria to survive unfavorable conditions on beewolf cocoons during larval hibernation and by the production of environmentally resistant antibiotics that remain stable on the cocoon as an efficient protection until emergence of the adult beewolf.

### Growth and morphological differentiation of beewolf symbionts on the cocoon

Species of the genus *Streptomyces* are well known for the production of numerous small molecules that are biologically active outside of the cell. Many of those substances are inhibitors of enzymes and cellular processes that act as antimicrobials [48,49,59,60], which help the producers to compete with antagonistic microorganisms or act as signaling molecules affecting gene transcription in co-occurring microbes [48,49]. In many species, the production of antimicrobial secondary metabolites has been found to be tightly linked to morphological differentiation into inactive spores [59,61-65]. In this context, the antimicrobial compounds may protect the cell’s metabolites from competitors during the vulnerable phase of re-allocation of the resources into spores. Prior studies of ‘*Candidatus* Streptomyces philanthi’ already proposed a morphological differentiation of the bacteria when applied to the surface of beewolf cocoons as a mechanism to survive the long period on the likely nutrient-limited cocoon surface during beewolf hibernation [58]. Our results provide support for this hypothesis and indicate that the symbiont population only grows within the first two days following cocoon spinning and subsequently undergoes morphological differentiation (Figures 1 and 3). Concordantly, two of the three analyzed housekeeping genes (*ftsZ* and *EF-Tu*) as well as the sporulation regulatory gene *whiB* show high expression levels during the first week on the cocoon, but then their expression is reduced to background levels (Figure 3a-c). *WhiB*, in particular, is known to be essential for early morphological differentiation as well as for the regulation of later sporulation gene transcription [66,67]. It closely interacts with *ftsZ,* which regulates septum formation in normal cell division as well as during sporulation [68,69]. Recent studies demonstrated that the expression of several regulatory *whi* genes, including *whiB*, is important for the correct timing of *ftsZ* and, as a result, for an optimal *FtsZ* protein level, which is essential for the formation of aerial biomass prior to morphological differentiation [68]. The early onset of *whiB* and *ftsZ* expression on the cocoon (already at day 0) suggests that at least some of the symbiont cells already initiate morphological differentiation prior to incorporation into the cocoon silk. This is supported by the observation that low amounts of antibiotics can already be detected in the antennal gland secretion of female beewolves within the brood cell (T. Engl, S. Koehler, and M. Kaltenpoth, unpublished data).

Surprisingly, *gyrB* showed a much slower decrease in expression levels after day 8 as compared to the other genes (Figure 3d), suggesting that gyrase may play an important role during the later stages of morphological differentiation. Concordantly, previous studies in *Bacillus subtilis*[70] and in *E. coli*[71-73] observed an inhibition of sporulation by gyrase inhibitors. Taken together, our gene expression analyses provide strong evidence for morphological differentiation of the beewolf symbionts into metabolically inactive spores about 1–2 weeks after cocoon spinning.

### Dynamics of antibiotic production and PKS gene expression

As observed for free-living *Streptomyces* strains under *in vitro* conditions [63,65,74], morphological differentiation coincided with antibiotic production in ‘*Ca*. S. philanthi’. The expression profile of a gene within the putative piericidin biosynthesis gene cluster (*pks1*) [Nechitaylo et al., unpubl. data, and ref. [90]] showed up-regulation between day 1 and day 4 after cocoon spinning, followed by down-regulation from day 8 onwards (Figure 2c). Accordingly, the highest amounts of antibiotics could be detected by GC-MS following the peak in antibiotic gene expression, i.e. between day 8 and 16 (Figure 2a). By producing antibiotic substances within the first two weeks after cocoon spinning, the symbionts establish a high concentration of antimicrobials on the surface of the cocoons. These compounds remain stable for months during hibernation, with an average total amount of 12.5 μg still present when the adult beewolf emerges. Even on nutrient-rich agar plates, this amount is high enough to effectively inhibit the growth of a wide range of soil fungi [40]. Thus, the stability of the antibiotic substances allows them to serve as an efficient long-term defense throughout the beewolf’s developmental phase.

### Stability of antibiotics mediates long-term antimicrobial protection for wasp offspring

In natural habitats, antibiotic compounds are subject to inactivation and degradation by different physicochemical and biological processes, e.g. photolysis, sorption, hydrolysis, and biodegradation [75-77]. Consequently, the stability varies greatly across compound classes and is strongly affected by abiotic and biotic conditions. While some substances are stable for months under aerobic conditions (e.g. fluorquinolones, [78,79]), others (e.g. sulphonamides) experience rapid degradation and are cleared from the environment within 2–3 weeks [80,81]. Polyene antibiotics, the class containing the piericidins, have been described as compounds which in solution are decomposed upon exposure to air, heat and light [82], while they show much higher stability (for up to several years) in dry state and in the absence of heat and light [83,84]. In the beewolf symbiosis, the localization of the piericidins on the cocoon surface in the dark and moderate climate of the subterranean brood cell may explain how these compounds can stably subsist in the environment for a prolonged period of time. The long-term stability of the antibiotic cocktail is crucial for an efficient protection of the beewolf offspring during hibernation, and it is therefore strongly selected for on the holobiont level of the interacting partners.

In addition to the absolute amount of antibiotics, the composition of the cocktail changes on the cocoon surface over time (Figure 2b, Additional file 1: Figure S1). PA1 is overrepresented in the first days after cocoon spinning, while its relative amount declines later on in favor of a higher proportion of PB1 and SC, suggesting differences in the timing of production as well as differential stability of the compounds. Possibly, PA1 is converted into PB1 by methylation of the hydroxyl group at the C16 position, which may explain the delayed increase and deterioration of PB1. The adaptive value of the changes in the antibiotic cocktail remains to be investigated. It is conceivable that the changing composition aids in preventing the emergence of resistant strains [85] or serves to efficiently ward off a succession of different soil fungi and bacteria [86].

### The importance of antibiotics for the wasp’s offspring

The stability of the symbiont-produced antimicrobial cocktail is essential for beewolves considering their brood care strategy. While social insects generally supply their progeny progressively with fresh food, mass provisioning is by far the most common strategy among solitary taxa [19,87]. In mass provisioning species, the adult insect supplies all of the food for each offspring prior to laying the egg and subsequently closes the brood cell. This strategy has the advantage that a closed brood cell is less prone to invasion by parasitoids, e.g. cuckoo wasps. However, the risk of pathogen infestation in subterranean nesting sites is severe, especially in combination with the storage of resources. In progressive provisioners such as social insect societies, this problem is counteracted by worker insects continuously cleaning off pathogens and contaminated materials from developing brood and nestmates [11-13], or by applying antimicrobial compounds to limit the spread of pathogens [9]. By contrast, mass-provisioning species like the European beewolf lack the possibility to continuously apply defensive chemicals or actively remove pathogens to protect their progeny. Therefore, the protection of the young with antimicrobial substances that are long lasting and resistant against environmental influences represents a particularly adaptive mechanism of parental care. In European beewolves, the symbiont-provided antibiotic treatment complements additional parental investments by the adult female to protect the offspring from pathogen infestation, such as embalming the prey bees with a postpharyngeal gland secretion to reduce fungal germination [5,88,89]. Altogether, these mechanisms ensure a successful completion of larval development for the beewolf progeny over their unpredictable developmental phase and serve as a reliable long term prophylaxis against pathogen attack.

It seems likely that long-term defensive strategies including symbiosis with antibiotic-producing microorganisms are more common among the large number of solitary insects that mass-provision their offspring in subterranean brood cells. However, the often unusual localization of protective symbionts as well as the inherent context-dependency of defensive symbioses hinder the detection of such associations. The increasing affordability of high-throughput sequencing technologies for microbial community analyses may at least partly ameliorate this problem by providing detailed insights into insect-associated microbial symbionts and uncovering candidates with putative protective functions.

## Conclusions

In the beewolf-*Streptomyces* mutualism, symbiont-produced antibiotic substances have previously been found to significantly enhance the survival probability of the wasp’s offspring [20]. Here we show that these compounds are produced only within a short period after larval cocoon spinning but subsequently remain stable on the cocoon surface for up to nine months of beewolf hibernation, thereby providing a reliable long-term protection for the developing beewolf larva against pathogens. Changes in the composition of the “antibiotic cocktail” over time indicate different production and degradation dynamics for the antibiotic compounds, which may aid to prevent the evolution of resistant microorganisms. The production of antibiotics on the cocoon coincides with morphological differentiation of the symbionts, which enables them to survive the inhospitable conditions during beewolf hibernation and to be successfully acquired from the cocoon surface by the next generation of emerging wasps. Thus, this symbiotic system appears to be finely tuned towards enhancing the efficiency of vertical symbiont transmission as well as providing long-term protection to the host offspring by warding off a broad range of pathogenic microorganisms. These findings provide new insights into the ecology of antibiotic production in the natural environment and may aid in exploring new strategies for the utilization of antibiotics in human medicine by using multi-drug combination therapies to counteract the increasing risk of resistant bacterial and fungal pathogens.

## Methods

### Beewolf rearing conditions

Female European beewolves, *Philanthus triangulum*, were obtained from a natural population in Berlin, Germany. The wasps were individually housed in observation cages [57] situated in the greenhouse (14 h day, 10 h night; 23°C (+/− 3°C fluctuation) and provided *ad libitum* with honeybees *(Apis mellifera)* as prey and honey as food for the adult females within the cage.

### Sample preparation

Male beewolf cocoons were obtained from the females’ nests at different time points after cocoon spinning (day 0, 1, 2, 4, 8, and 16 after cocoon spinning). The cocoons were longitudinally cut to create an opening through which the larva was carefully removed using forceps. After dissection, the cocoon samples were directly stored at −80°C. Additionally, some cocoons with larvae were left to complete their development, and the cocoons were collected directly after the adult beewolf emerged from the cocoon. Some of these individuals developed directly within about four weeks (“emergence without hibernation”), while others went into diapause and emerged in the following spring (“emergence after hibernation”). All cocoon samples for emergence were transferred from the observation cages into open 1.5 ml Eppendorf cups approximately one week after cocoon spinning, placed in a box with moist sand and stored in an incubator at 26°C. Cocoons of individuals that developed directly and emerged within the same year were dissected immediately after emergence and frozen at −80°C. Individuals that had not entered the pupal stage four weeks after cocoon spinning were assumed to have entered diapause. These cocoons were transferred to the fridge and stored at 6°C for eight months over winter to mimic hibernating conditions. Subsequently, the samples were returned to the incubator at 26°C to induce pupation, so the cocoons in this group were about 9–10 months old upon sampling (eight months of diapause plus 4–8 weeks of development). After emergence of the adult beewolves, cocoons were stored at −80°C for subsequent analyses.

### Methanol extraction

Frozen (−80°C) beewolf cocoons (n =157) were thawed on ice. The cocoons were transferred with forceps to GC-MS vials (1.5 ml vials, 6 mm jar opening, CZT, Kriftel, Germany), and 1 ml of methanol (>99.9%, Roth, Karlsruhe, Germany) was added to each sample. Forceps were cleaned in methanol and hexane in between samples to prevent contamination. Finally, the samples were placed on a shaker (Heidolph Vibramax 100) for 1 h at 350-400 rpm. After extraction, cocoons were taken out of the GC-MS vials, placed on a tissue to drain methanol and finally transferred to clean 1.5 ml cups (Eppendorf). Cocoon samples were placed under the hood for approximately 1 h to evaporate the remaining methanol for molecular analyses. Methanol extracts were stored at −20°C for subsequent GC-MS analysis.

### GC-MS analysis of antibiotics

Methanol extracts were taken out of the freezer and evaporated to dryness under a gentle stream of Argon. Antibiotics were re-suspended in 50 μl of methanol and transferred to a 150 μl GC-μ-vial (CZT, Kriftel, Germany) for GC-MS analysis. An aliquot of 1 μl of each sample was injected into a Varian 450GC gas chromatograph coupled to a Varian 240MS mass spectrometer (Agilent Technologies, Böblingen, Germany) using a split/splitless injector at 250°C with the purge valve opened after 60 s. The GC was equipped with a DB5-MS capillary column (30 m × 0.25 mm diameter, film thickness: 0.25 μm, Agilent Technologies) and programmed from 150 to 300°C at 5°C/min with a 1min. initial isothermal and a 5min. final isothermal hold. Helium was used as carrier gas, with a constant flow rate of 1 ml/min. Mass spectra were recorded using electron ionization (EI-MS). Data acquisition and quantifications were achieved with MS Workstation Version 6.9.3 Software (Agilent Technologies). The five most abundant compounds on beewolf cocoons were quantified, i.e. streptochlorin (SC), piericidin A1 (PA1), piericidin B1 (PB1), piericidin A5 (PA5) and piericidin C1 (PC1) [40]. A dilution series (500–0.1ppm) of commercially available piericidin A1 was used as an external calibration standard for the four different piericidin derivatives, assuming similar ionization efficiencies based on the high structural similarity. For quantification of streptochlorin, we used a dilution series of a synthesized streptochlorin standard (for the synthesis of streptochlorin see Additional file 2: Supplementary methods). The peaks were identified by comparison of their mass spectra with the standard spectra or with published reference spectra [40], and peak areas were automatically integrated using the MS Workstation Software. Finally, the success of this integration was controlled manually for every peak.

### DNA/RNA extraction

After methanol extraction, the dried beewolf cocoons were used for nucleic acid extraction. The cocoons were homogenized in liquid nitrogen followed by DNA/RNA extraction using the Epicentre MasterPure™ DNA extraction kit (Epicentre Technologies, Madison, USA). The kit is based on a precipitation reaction and can be used for combined DNA/RNA extraction. All solutions not included in the kit (TE buffer, water, 70% EtOH, Isopropanol) were prepared from RNase-free stock solutions. The protocol was adjusted as follows: the lysozyme treatment was omitted, and all centrifugation steps were done at room temperature. Finally, the DNA pellet was resuspended in 100 μl RNase-free low-TE buffer (1 mM Tris/HCl, 0.01 mM EDTA). Each sample was partitioned in two aliquots with 50 μl each and stored at −80°C.

### Quantification of *Streptomyces* population size and gene expression

Quantitative real-time PCR (qPCR) with diagnostic primer pairs was used to quantify the population size (16S rRNA gene copy number) and the expression of housekeeping (*gyrB, EF-Tu* and *ftsZ*), sporulation (*whiB*) and antibiotic genes (*pks1*) of ‘*Ca*. S. philanthi’. Primers were obtained from the literature (16S and *gyrB*: [58]) or designed based on available whole genome shotgun sequencing data (Nechitaylo et al., unpubl. data) using Primer3 ( http://primer3.sourceforge.net; Table 1). The *pks1* gene is localized within the cluster that is predicted to be involved in piericidin biosynthesis, based on *in silico* prediction as well as by the high similarity to the published piericidin gene cluster of *Streptomyces piomogenus var. hangzhouwanensis*[90]. The PCR conditions for each primer pair were optimized using gradient PCR with a template extracted from female beewolf antennae, and specificity was confirmed by gel electrophoresis and sequencing.

**Table 1:** **Primers used for PCR and quantitative PCR amplification of ‘*Ca*. S. philanthi’ genes**

**Primer**	**Sequence (5′-3′)**	**Direction**	**Ampl icon length**	**Reference**
Strep_phil_fwd3mod	TGGTTGGTGGTGGAAAGC	Fwd	135	Kaltenpoth et al., 2010
Strep_16S_rt_rev	GTGTCTCAGTCCCAGTGTG	Rev		Kaltenpoth et al., 2010
PKS1-SPT-F1	TCTTCCGACAGTCGATAGCC	Fwd	139	This study
PKS1_SPT-R1	GAGATCATGACGGCGAAGAG	Rev		This study
Strep_phil_EF-Tu_rt_fwd	CGACTACACGCACAAGAAG	Fwd	108	This study
Strep_phil_EF-Tu_rt_rev	CACGGACGGGATGTACTC	Rev		This study.
Strep-phil-gyrB-rt_fwd	CGCCAACACGATCCACAC	Fwd	115	Kaltenpoth et al., 2010
Strep-phil-gyrB-rt_rev	GTCCTTCTCCCGCAGCAG	Rev		Kaltenpoth et al., 2010
FtsZ-Ptr_F1	GACCGACTGCTGTCCATTTC	Fwd	136	This study
FtsZ-Ptr_R1	CGAAGTCCAGGTTGATCAGG	Rev		This study
whiB(189)-F1	CGAGCTGTTCTTCCCCATC	Fwd	104	This study
whiB(189)-R1	ACTGCAGGCACTCCTCCAT	Rev		This study

To establish a standard curve for each gene of interest, DNA extracts of female beewolf antennae were thawed and used for PCR amplification. Amplification was performed on a VWR Gradient Thermocycler (UnoCycler, VWR, Darmstadt, Germany) in a total reaction volume of 12.5 μl, containing: 1 μl template DNA, 1×PCR buffer (key buffer, Tris/HCl, (NH_4_)_2_SO_4_ and 0.1% Tween 20), 2.5 mM MgCl_2_ (including the 1.5 mM MgCl_2_ in the buffer), 240 μM dNTPs, 0.8 μM of each primer and 0.5U *Taq* DNA-Polymerase (VWR, Darmstadt, Germany). Cycle parameters were as follows: an initial denaturation step at 94°C for 3 min, 35 cycles of 94°C for 40 s, 65°C for 40 s and 72°C for 40 s, and a final extension step of 72°C for 4 min. PCR success was verified by gel electrophoresis using a GelRed™ (Biotium, Hayward, USA) stained 1.5% TBE agarose gel (150V, 30 min). The documentation of gel pictures was conducted using GeneSnap image acquisition software (GeneSnap 7.09.06, Syngene, Camebridge, United Kingdom). Positive bands were excised from the gel and purified using PeqGold MicroSpin Gel extraction kit (Peqlab, Erlangen, Germany). The final DNA concentration of the purified PCR-product was obtained from NanoDrop measurements (ND1000 photo-spectrometer, PeqLab, Erlangen, Germany). Finally, the DNA samples were diluted to 1 ng/μl with RNase free water, and a serial dilution of 10^-1^ to 10^-8^ ng/μl was established to serve as a standard for qPCR quantification.

QPCR was used to quantify the *Streptomyces* population size on the surface of beewolf cocoons. Thawed DNA extracts of whole beewolf cocoons were used for qPCR amplification with ‘*Ca*. S. philanthi’ 16S rRNA gene-specific primers (Table 1) in a total reaction volume of 25 μl containing the following reagents: 6.5 μl RNase free water and 12.5 μl SYBR-Mix (Rotor-Gene SYBR Green RT-PCR kit, Qiagen, Hilden, Germany); 2.5 μl of each primer (10 μM), 1 μl template. Quantitative PCR was performed on a Rotor-Gene Q Cycler (Qiagen, Germany) using the following cycle parameters: 95°C for 10 min, 45 cycles of 95°C for 15 s, 60°C for 30 s, 72°C for 20 s, and a final melting curve analysis was performed by increasing the temperature from 72 to 95°C with 1°C gain. Based on the standard curve, the total amount of DNA was calculated based on the qPCR threshold values using the absolute quantification method [91,92].

For quantification of the antibiotic gene expression as well as the expression of the housekeeping and sporulation genes, RNA extracts of whole beewolf cocoons were thawed and used for reverse transcription to obtain cDNA using the QuantiTect reverse transcription kit (Qiagen, Germany) and the specific primer pairs according to the manufacturer’s instructions. Subsequently, these cDNA samples were used for qPCR amplification with the same primers, using the same amplification conditions as indicated above.

### Statistical analysis

Statistical analyses were performed using SPSS 17.0 Software (IBM, New York, USA). The amount of antibiotics estimated from GC-MS analysis as well as the symbiont 16S copy numbers and expression levels of housekeeping, antibiotic production, and sporulation genes estimated by qPCR were compared across different time points using ANOVA with Tukey posthoc measures. Additionally, changes in the composition of the antibiotic cocktail over time were analyzed based on the most abundant and consistently detected compounds, i.e. streptochlorin, piericidin A1 and piericidin B1. Omitting the compounds that were not consistently detected (PC1, PA5) is an approach that is conservative with regard to the hypothesis tested, i.e. that the composition of the cocktail changes over time. Single peak areas were translated into relative peak areas and subsequently log-ratio transformed using the Aitchison transformation [93]. Finally, a discriminant analysis was used to elucidate differences in the composition of the antibiotic cocktail on beewolf cocoons over time (Figure 2b, Additional file 1: Figure S1).

## Competing interests

The authors declare that they have no competing interests.

## Authors’ contributions

SK and MK conceived of the study. SK performed sample collection, experiments and analyses. SK and MK wrote the manuscript. JD performed the synthesis of streptochlorin for the quantification. All authors read and approved the final manuscript.

## Supplementary Material

Additional file 1: Figure S1 Canonical discriminant functions. Click here for file

Additional file 2 Supplementary methods. Click here for file
