# Supplementary figures and images for: Dynamics of symbiont-mediated antibiotic production reveal efficient long-term protection for beewolf offspring
Source: Front Zool. 2013 Jan 31;10:3. doi: 10.1186/1742-9994-10-3 (PMC3599432; doi:10.1186/1742-9994-10-3)

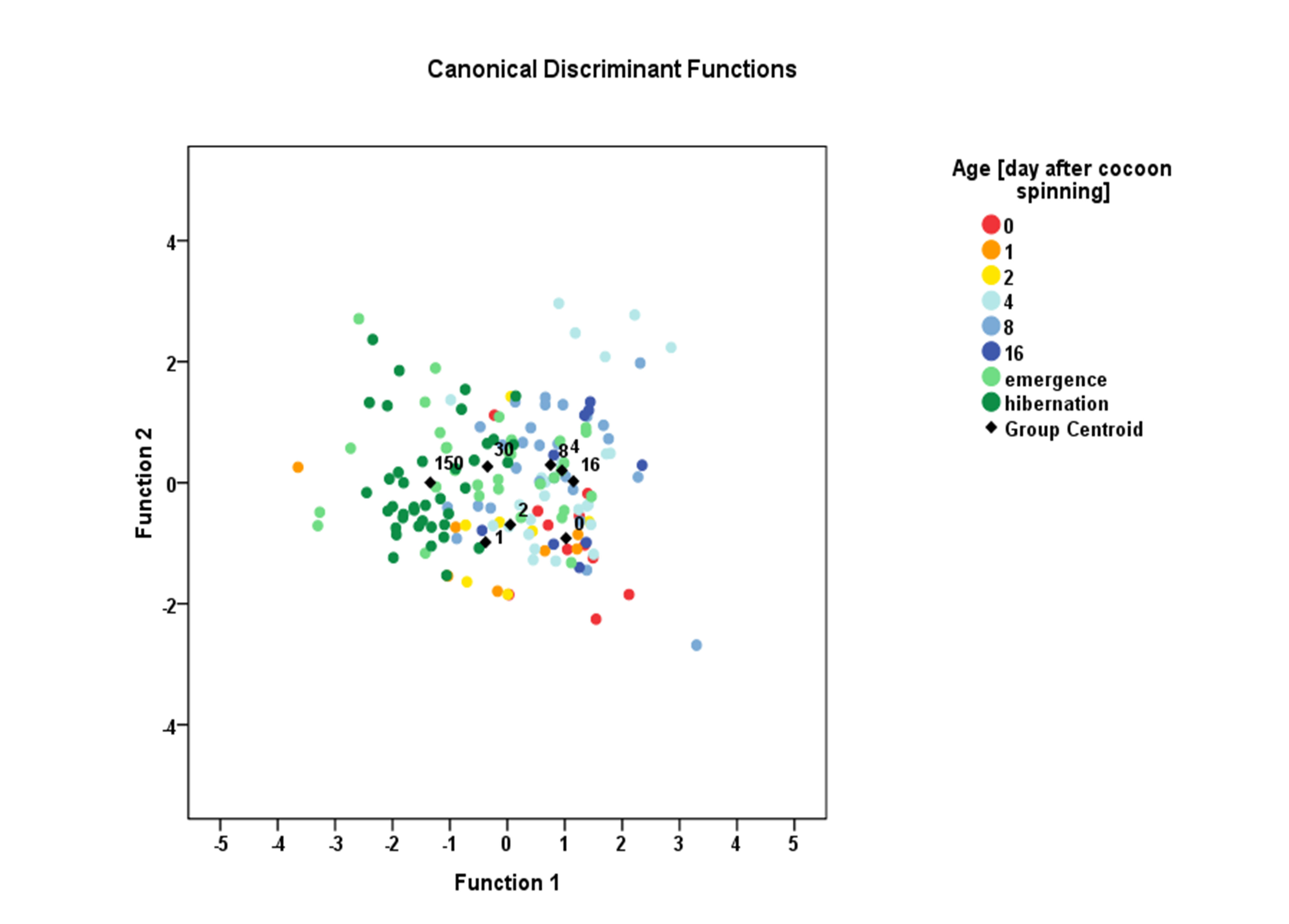

Supplement: Additional file 1: Figure S1 — Canonical discriminant functions. [file 1742-9994-10-3-S1.jpeg]
