# Supplementary material for: Dynamics of symbiont-mediated antibiotic production reveal efficient long-term protection for beewolf offspring
Source: Front Zool. 2013 Jan 31;10:3. doi: 10.1186/1742-9994-10-3 (PMC3599432; doi:10.1186/1742-9994-10-3)
Supplement: Additional file 2 — Supplementary methods. [file 1742-9994-10-3-S2.docx]

**Supplementary methods**

**Synthesis of Streptochlorin**

Commercially available chemicals were used without further purification, and solvents were dried as follows: dichloromethane and DMPU were dried by storage over molecular sieves 4Å for several days prior to use, dry methanol and THF (Extra Dry, AcroSeal^®^, Acros Organics), and absolute DMF (Fluka) were used as received. All reactions were carried out under an argon atmosphere. The preparative column chromatography (flash chromatography) was performed on Merck Kieselgel 60 (230-400 mesh), and TLC analysis on commercial Merck Kieselgel 60 F_254_ plates. Melting points were determined on a Büchi Melting Point apparatus B-540 and are uncorrected. ^1^H-NMR and ^13^C-NMR spectra were measured on a Bruker Avance DRX 500, AV-500, and 400 NMR spectrometers. Chemical shifts are reported in ppm downfield from TMS. Structures of all compounds were confirmed by signal assignment based on standard COSY, HSQC and HMBC spectra. HRMS and MS (EI) measurements (direct input mode) were performed with a MasSpec 2 instrument (Micromass) in positive ion mode using 70eV ionization energy. ESI-MS measurements were performed with a UltiMate 3000 UPLC (Dionex) coupled to an Orbitrap XL (Thermo-Fisher) equipped with an electrospray (ESI) source.

***Scheme 1***: TsCl = *p-*toluenesulfonyl chloride; TBAB = tetra-*n*-butylammonium bromide; TIPSOTf = triisopropylsilyl triflate; LiHMDS = lithium hexamethyldisilazane; DMPU = *N,N´-*dimethylpropylene urea; NCS = *N*-chlorosuccinimide; TBAF = tetra-*n*-butylammonium fluoride.

**1-(Toluene-4-sulfonyl)-1*H*-indole-2-carbaldehyde (1)**

According to a reported procedure [[1](#_ENREF_1)], aqueous 30% NaOH (90 ml, 0.675 mol), tosyl chloride (5.01 g, 26.28 mmol) and tetra-*n*-butylammonium bromide (0.81 g, 10 mol%) were added to a suspension of indole-3-carboxaldehyde (3.63 g, 25.0 mmol) in 90 ml of benzene. After stirring vigorously for 30 min, the layers were separated and the aqueous layer was extracted with benzene (1× 50 ml). The combined organic layers were extracted with water, 10% aq. citric acid, and brine (1× 20 ml each), dried with MgSO_4_, and, after addition of charcoal, passed through a short column of silica gel. The column was washed with 100 ml of benzene and the collected fractions were evaporated under reduced pressure. The crude product was purified by crystallization from a 2:1 *n*-hexane-benzene mixture affording 6.752 g (90 %) of the aldehyde as an off-white crystalline solid.

^1^H NMR (500 MHz, CDCl_3_): δ = 2.36 (s, 3H), 7.29 (m, 2H, *J* = 8.5 Hz), 7.35 (ddd, 1H, *J* = 7.8, 7.3 and 1.1 Hz), 7.40 (ddd, 1H, *J* = 8.3, 7.3 and 1.4 Hz), 7.85 (m, 2H, *J* = 8.5 Hz), 7.95 (ddd, 1H, *J* = 8.3, 1.1 and 0.7 Hz), 8.23 (s, 1H), 8.25 (ddd, 1H, *J* = 7.8, 1.4 and 0.7 Hz), 10.09 (s, 1H). ^13^C NMR (125 MHz, CDCl_3_): δ = 21.65, 113.24, 122.35, 122.59, 125.04, 126.28, 126.30, 127.22 (2C), 130.31 (2C), 135.22, 134.34, 136.23, 146.16, 185.32.

**3-(Oxazol-5-yl)-1-(toluene-4-sulfonyl)-1*H*-indole (2) and 3-(oxazol-5-yl)-1*H*-indole (3)**

The compounds were prepared according to a reported procedure [[2](#_ENREF_2)] with minor modifications.

*Method A:* A solution of 300 mg of the aldehyde **1** (1 mmol), 215 mg of TOSMIC (1.1 mmol), and 155 mg of anhydrous K_2_CO_3_ (1.1 mmol) was refluxed in 10 ml of methanol for 1 hour. The reaction mixture was allowed to cool down to room temperature, poured into brine (50 ml), and extracted with EtOAc (4× 20 ml). The combined organic extracts were dried with MgSO_4_ and evaporated. The residue was purified by column chromatography on silica gel (25-50% EtOAc/*n*-hexane) to furnish 164 mg (48 %) of the N-Ts-derivative **2** as off-white crystals, and 14 mg (8 %) of the deprotected product **3** as pale beige crystals. [Due to the content of other byproducts and similar R_F_ values, the purification of both products **2** and **3** (R_F_ = 0.26 and 0.20 in 50% EtOAc/hexane) was quite tedious and led only to an incomplete separation, providing thus diminished yields. Therefore, the pre-purified reaction mixture was used directly in the next step (*Method B*)].

*Method B:* Following *Method A*, 3.0 g of the aldehyde (10 mmol), 2.15 g of TOSMIC (11 mmol), and 1.52 g of K_2_CO_3_ were refluxed for 3 hours. After the workup, the reaction mixture was passed through a short column of silica gel and eluted with 35% EtOAc/*n*-hexane. The fractions containing products **2** and **3** were collected and evaporated to afford 2.63 g of yellow oil.
The crude material was dissolved in 90 ml of a 2:1 mixture of THF/MeOH, 7.59 g of Cs_2_CO_3_ (3 equiv.) [[3](#_ENREF_3)] was then added, and the mixture was heated to 60 °C for 2 hours. After cooling to room temperature, the reaction mixture was poured into water (200 ml) and extracted 4× with 50 ml of EtOAc. The combined organic fractions were washed once with 5% aq. citric acid (50 ml), once with brine (50 ml), dried with MgSO_4_, and evaporated under reduced pressure. The crude mixture was purified by column chromatography on silica gel (0-2% MeOH/CHCl_3_) to provide 1.176 g (64 % based on the starting aldehyde) of the 3-(oxazol-5-yl)-1*H*-indole **3**.

**2**: ^1^H NMR (500 MHz, CDCl_3_): δ = 2.34 (s, 3H), 7.24 (m, 2H, *J* = 8.5 Hz), 7.35 (ddd, 1H, *J* = 7.8, 7.4 and 1.1 Hz), 7.38 (s, 1H), 7.41 (ddd, 1H, *J* = 8.2, 7.4 and 1.2 Hz), 7.77 (d, 1H, *J* = 7.8 Hz), 7.81 (m, 2H, *J* = 8.5 Hz), 7.92 (s, 1H), 7.95 (s, 1H), 8.04 (d, 1H, *J* = 8.2 Hz). ^13^C NMR (125 MHz, CDCl_3_): δ = 21.60, 110.91, 113.88, 120.52, 122.28, 123.06, 124.03, 125.53, 126.89, 126.96 (2C), 130.07 (2C), 134.86, 135.10, 145.43, 145.67.

**3**: ^1^H NMR (500 MHz, *d_6_*- acetone): δ = 7.19 (ddd, 1H, *J* = 7.9, 7.0 and 1.2 Hz), 7.23 (ddd, 1H, *J* = 8.1, 7.0 and 1.3 Hz), 7.38 (s, 1H), 7.52 (m, 1H, *J* = 8.1 Hz), 7.79 (d, 1H, *J* = 2.4 Hz), 7.91 (m, 1H, *J* = 7.9 Hz), 8.11 (s, 1H), 10.71 (bs, 1H). ^13^C NMR (125 MHz, *d_6_*- acetone): δ = 105.56, 112.83, 119.95, 120.45, 121.25, 123.30, 123.94, 125.07, 137.72, 149.11, 149.97.

**3-(Oxazol-5-yl)-1-(triisopropylsilyl)-1*H*-indole (4)**

The bulky triisopropylsilyl (TIPS) group was chosen to protect the indole moiety, before the next step, because *N*-TIPS substitution is known to prevent deprotonation at C(2). Thus, contrary to some other silyl groups, TIPS group is also resistant toward rearrangements upon treatment with strong bases.

To a stirred solution of 3-(oxazol-5-yl)-1*H*-indole **3** (925 mg, 5.02 mmol) in 25 ml of anhydrous DMF was slowly added 60% dispersion of NaH in mineral oil (220 mg, 5.5 mmol) at 0 °C. After 20 minutes, 1.49 ml of triisopropylsilyl triflate (5.52 mmol) was added dropwise and the reaction mixture was left to stir for additional 15 minutes at 0 °C, and then at room temperature for 1 hour. The reaction mixture was quenched with 5 ml of saturated aq. NaHCO_3_, poured into a 1:1 mixture of brine and water (150 ml), and extracted 4× with 50 ml of 25% EtOAc/hexane. The combined organic fractions were dried with Na_2_SO_4_, evaporated, and the crude product was purified by column chromatography on silica gel (0-20% EtOAc/*n*-hexane) affording thus 1.674 g (98 %) of the title compound **4** as colorless crystals.

Mp 65.5 °C. ^1^H NMR (500 MHz, CDCl_3_) [Similarly poor resolution of the aromatic proton signals was observed when spectra measured either in *d_6_*-acetone or *d_6_*-benzene]: δ = 1.17 (d, 18H, *J* = 7.5 Hz), 1.75 (septet, 3H, *J* = 7.5 Hz), 7.24-7.26 (m, 2H), 7.30 (s, 1H), 7.54-7.56 (m, 1H), 7.59 (s, 1H), 7.83-7.85 (m, 1H), 7.89 (s, 1H). ^13^C NMR (125 MHz, CDCl_3_): δ = 12.87 (3C), 18.07 (6C), 107.41, 114.32, 119.52, 119.73, 120.93, 122.47, 127.41, 129.08, 141.34, 147.94, 148.76. MS (EI); *m/z* (%): 340 (100) [M^+^], 297 (55), 255 (12), 227 (7), 168 (6), 115 (11). HRMS *m/z* 340.1956 (C_20_H_28_N_2_OSi requires 340.1971).

**3-(4-Chlorooxazol-5-yl)-1-(triisopropylsilyl)-1*H*-indole (5)**

The procedure for regioselective chlorination of the oxazole **4**, described herein, has been adapted from a method developed for iodination of oxazoles at C(4) [[4](#_ENREF_4)]. An approach based on the use of DMF as a participating solvent [[5](#_ENREF_5)] was also tested but gave only 17 % yield.

A solution of the oxazole **4** (1.221 g, 3.585 mmol) in a mixture of 10 ml of dry THF and 10 ml of dry DMPU was cooled to -70 °C, and 1M solution of lithium hexamethyldisilazane in THF (4.3 ml, 1.2 equiv.) was added dropwise. The reaction mixture was allowed to warm to -40 °C within 1 hour and then cooled again to -80 °C. A solution of 708 mg of N-chlorosuccinimide (5.3 mmol, 1.48 equiv.) in 10 ml of dry DCM was added slowly at this temperature via syringe. The resulting orange slurry was stirred (with occasional shaking) at -60 to -40 °C for an additional 1 hour, quenched at -40 °C by addition of 20% aq. solution of NH_4_Cl (2 ml), and allowed to warm to room temperature. The mixture was poured into a vigorously stirred mixture of 200 ml of water, containing 0.5 g of Na_2_S_2_O_3_, and 50 ml of 10% EtOAc/*n*-hexane. The organic layer was separated and the aqueous phase was extracted 3× with 10% EtOAc/*n*-hexane. The combined organic fractions were dried with MgSO4 briefly and evaporated to give 1.68 g of brown oil which was purified by column chromatography on silica gel (0-5% EtOAc/*n*-hexane). The pure product (685 mg, 49 % yield) was obtained as yellow viscous oil which solidifies upon standing to give ochre crystals.

Mp 84-86 °C. ^1^H NMR (400 MHz, C_6_D_6_): δ = 0.96 (d, 18H, *J* = 7.5 Hz), 1.42 (septet, 3H, *J* = 7.5 Hz), 7.03 (s, 1H), 7.26 (ddd, 1H, *J* = 7.9, 7.1 and 1.4 Hz), 7.31 (ddd, 1H, *J* = 7.7, 7.1 and 1.1 Hz), 7.48 (dd, 1H, *J* = 7.9 and 1.1 Hz), 7.96 (s, 1H), 8.27 (dd, 1H, *J* = 7.7 and 1.4 Hz),. ^13^C NMR (100 MHz, C_6_D_6_): δ = 12.93 (3C), 18.04 (6C), 106.62, 114.37, 121.64, 121.71, 122.71, 123.18, 128.59, 130.55, 141.29, 143.48, 147.80. MS (EI); *m/z* (%): 374 (100) [M^+^], 331 (56), 289 (13), 261 (8), 137 (12), 115 (17). HRMS *m/z* 374.1582 (C_20_H_27_^35^ClN_2_OSi requires 374.1581).

**3-(4-Chlorooxazol-5-yl)-1*H*-indole – Streptochlorin (6)**

A solution of the TIPS-protected chlorooxazole **5** (684 mg, 1.82 mmol) in 15 ml of dry THF was treated with 1M solution of TBAF in THF (3.7 ml, 2 equiv.) at room temperature for 1 hour. The reaction mixture was poured into a 1:1 mixture of saturated aq. NaHCO_3_ and brine (50 ml), and extracted with dichloromethane (4× 15 ml). The combined organic fractions were washed 1× with water (10 ml) and 1× with brine (10 ml), dried with MgSO_4_, and evaporated under reduced pressure to give light brown oil. Column chromatography on silica gel (10-50% EtOAc/*n*-hexane) afforded 336 mg (85 %) of pure streptochlorin as pale beige crystals. An analytical sample was obtained by further crystallization from benzene.

LC-MS data, including MS-MS of the [M+H]^+^ ion, and NMR spectra (*d_4_*-MeOH) were in accordance with those reported previously [[6](#_ENREF_6)].

Mp 170.8 °C. ^1^H NMR (500 MHz, *d_4_*-MeOH): δ = 7.15 (ddd, 1H, *J* = 8.1, 7.0 and 1.0 Hz), 7.22 (ddd, 1H, *J* = 8.1, 7.0 and 1.0 Hz), 7.46 (d, 1H, *J* = 8.1 Hz), 7.83 (s, 1H), 8.00 (d, 1H, *J* = 8.1 Hz), 8.18 (s, 1H). ^1^H NMR (500 MHz, *d_6_*-acetone): δ = 7.19 (ddd, 1H, *J* = 8.0, 6.9 and 0.9 Hz), 7.25 (ddd, 1H, *J* = 8.1, 6.9 and 1.0 Hz), 7.55 (d, 1H, *J* = 8.1 Hz), 7.97 (bd, 1H, *J* = 2.3 Hz), 8.04 (d, 1H, *J* = 8.0 Hz), 8.23 (s, 1H), 10.86 (bs, 1H). ^13^C NMR (125 MHz, *d_6_*-acetone): δ = 103.5, 112.87, 121.12, 121.48, 121.78, 123.62, 125.20, 125.50, 137.25, 144.17, 149.44. MS (EI); *m/z* (%): 218 (100) [M^+^], 189 (13), 183 (9), 163 (11), 155 (36), 144 (16), 128 (17). HRMS *m/z* 218.0251 (C_11_H_7_^35^ClN_2_O requires 218.0247).

**References**

1. Waser J, Gaspar B, Nambu H, Carreira EM: **Hydrazines and azides via the metal-catalyzed hydrohydrazination and hydroazidation of olefins.** *J Am Chem Soc* 2006, **128:**11693-11712.

2. Chakrabarty M, Basak R, Harigaya Y, Takayanagi H: **Reaction of 3/2-formylindoles with TOSMIC: formation of indolyloxazoles and stable indolyl primary enamines.** *Tetrahedron* 2005, **61:**1793-1801.

3. Bajwa JS, Chen GP, Prasad K, Repic J, Blacklock TJ: **Deprotection of N-tosylated indoles and related structures using cesium carbonate.** *Tetrahedron Lett* 2006, **47:**6425-6427.

4. Vedejs E, Luchetta LM: **A method for iodination of oxazoles at C-4 via 2-lithiooxazoles.** *J Org Chem* 1999, **64:**1011-1014.

5. Li B, Buzon RA, Zhang Z: **Syntheses of 4,5-disubstituted Oxazoles via regioselective c-4 bromination.** *Org Process Res Dev* 2007, **11:**951-955.

6. Kroiss J, Kaltenpoth M, Schneider B, Schwinger MG, Hertweck C, Maddula RK, Strohm E, Svatos A: **Symbiotic streptomycetes provide antibiotic combination prophylaxis for wasp offspring.** *Nat Chem Biol* 2010, **6:**261-263.

**Supplementary figures**

**Fig.S1:** Multivariate discriminant analysis of the three most abundant antibiotics on beewolf cocoons (SC, PA1 and PB1) based on GC-MS data from Fig.2a (Wilks´ Lambda: 0.432, Chi2: 126.4, df=21, p<0.001).
